# Functional Characterization of Loss of *RNF43* Reveals Neuronal Defects in a *Caenorhabditis elegans* Model

**DOI:** 10.3390/ijms27104509

**Published:** 2026-05-18

**Authors:** Kaan Sonmez, Sinem Güzel, Hasan Huseyin Kazan, Burcu Ekim, Cem Kaya, Zafer Turkyilmaz, Ramazan Karabulut, Mehmet Ali Ergun

**Affiliations:** 1 Department of Pediatric Surgery, Faculty of Medicine, Gazi University, 06560 Ankara, Türkiye; kaans@gazi.edu.tr (K.S.); cemkaya@gazi.edu.tr (C.K.); zafert@gazi.edu.tr (Z.T.); ramazank@gazi.edu.tr (R.K.); 2 Rare Disease Laboratory, School of Life and Natural Sciences, Abdullah Gul University, 38040 Kayseri, Türkiye; sinemzeynepguzel@gmail.com; 3 Department of Medical Biology, Gülhane Faculty of Medicine, University of Health Sciences, 06010 Ankara, Türkiye; hasanhuseyinkazan@gmail.com; 4 Laboratory Animals Raising and Experimental Research Center (GUDAM), Gazi University, 06560 Ankara, Türkiye; bekim@gazi.edu.tr; 5 Department of Medical Genetics, Faculty of Medicine, Gazi University, 06560 Ankara, Türkiye

**Keywords:** RNF43, Wnt signaling, microcephaly, neurodevelopment, *Caenorhabditis elegans*

## Abstract

Ring finger protein 43 (*RNF43*) encodes a transmembrane E3 ubiquitin ligase that negatively regulates canonical Wnt signaling and is classically associated with serrated polyposis syndrome and colorectal cancer. In this study, regarding a homozygous truncating *RNF43* variant (NM_001305545.1:c.1906C>T; p.Gln636Ter) in a patient segregating with a severe neurodevelopmental phenotype characterized by developmental delay, neonatal hypotonia, recurrent seizures, progressive microcephaly, and bilateral optic atrophy, the loss of polarity defective 1 (*plr-1*), an ortholog of *RNF43*, was modeled in *Caenorhabditis elegans* and the phenotype was primarily characterized. The results demonstrated that loss of the *plr-1* disrupted gamma aminobutyric acid (GABA)ergic axon organization, reduced locomotor speed calculated from 60 s recordings, and altered developmental growth. These findings expand the phenotypic spectrum of RNF43 and support a dosage-dependent developmental role.

## 1. Introduction

Ring finger protein 43 (RNF43), a transmembrane E3 ubiquitin ligase, acts as a negative regulator of canonical Wnt signaling. RNF43 regulates Wnt signaling by ubiquitinating Frizzled receptors and promoting their internalization and degradation. Through this mechanism, RNF43 functions as a critical regulator of Wnt signaling intensity and spatial distribution during development [1,2,3].

The Wnt signaling pathway is highly conserved across species and regulates numerous biological processes, including embryonic development, stem cell maintenance, tissue homeostasis, and tumorigenesis [4,5]. Dysregulation of Wnt signaling has been implicated in multiple human diseases, particularly cancer [6]. Germline heterozygous variants in *RNF43* have strongly been associated with serrated polyposis syndrome and increased risks of colorectal cancer [7,8]. Beyond tumor biology, Wnt signaling plays an essential role in the development of nervous system. It regulates neural progenitor proliferation, cortical patterning, neuronal differentiation, axon guidance, and synaptic development [9]. Alterations in Wnt signaling have been implicated in several neurodevelopmental disorders, including microcephaly, epilepsy, and developmental delay [10].

Studies in model organisms have provided important insights into the role of Wnt signaling in neuronal development. In *Caenorhabditis elegans* (*C. elegans*), Wnt signaling regulates neuronal polarity, axon guidance, and synaptic organization [11,12]. The worm ortholog of *RNF43*, *plr-1*, has been shown to regulate the localization and signaling of Wnt receptors, indicating that plr-1 acts as a conserved regulator of Wnt signaling [11].

Despite the well-established role of RNF43 in tumor predisposition, the developmental consequences of biallelic *RNF43* variants remain largely unexplored. In the present study, we describe a consanguineous family carrying a homozygous truncating *RNF43* variant associated with severe neurodevelopmental manifestations. To investigate the functional consequences of RNF43 loss, we performed functional analyses using the *C. elegans* ortholog *plr-1*. Our findings provide evidence that disruption of *RNF43/plr-1* may affect neuronal organization and locomotor behavior, suggesting a previously underrecognized role of *RNF43* in neurodevelopment.

## 2. Results

**Figure 1:**
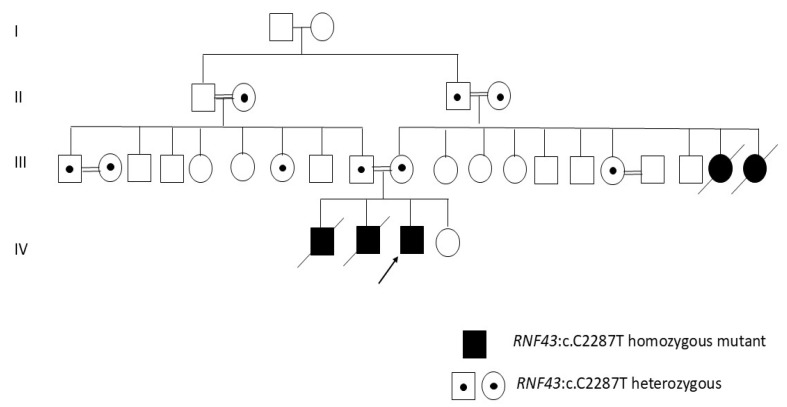
Pedigree of the family.

In the present study, the phenotype of a male patient with progressive microcephaly, frequent seizures, neonatal hypotonia, bilateral optic atrophy, and global development delay, which were also recorded for the death of two siblings, was attempted to be investigated in *C. elegans* model. For molecular diagnosis, whole-exome sequencing (WES) was performed by Twist Exome 2.0 Panel (Twist Biosciences, South San Francisco, CA, USA) and Illumina Next550 System (Illumina Inc., San Diego, CA, USA). According to variant analyses and familial segregation results (Figure 1), NM_001305545.1:c.1906C>T; p.Gln636Ter in the *RNF43* gene, putatively causing loss of protein function, was the only variant that may explain the genetic background of the disease.

Owing to relatively easy manipulation and cost performances, we have turned our interest in *C. elegans* to investigate the effects of the loss of protein on neurons, mobility, and neuronal morphology. Human *RNF43* is an ortholog of *C. elegans plr-1* gene. Similar to human RNF43, *C. elegans* plr-1 protein contains a zinc finger domain located between 317 and 358 aa, with a protein of 487 amino acids. Hence, we obtained *plr-1*(*tm2957*) mutants.

**Figure 2:**
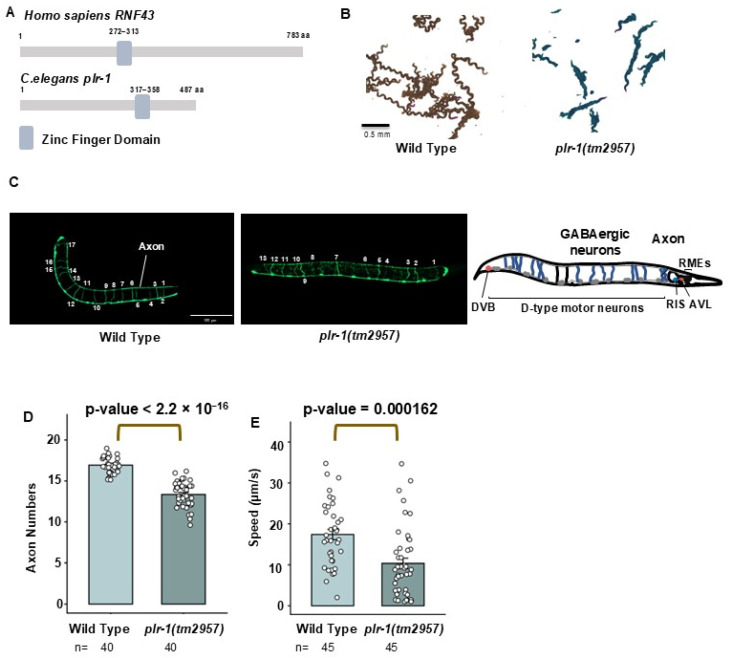
*C. elegans plr-1* mutants displaying locomotion and axonal defects. (**A**) Domain organization of human *RNF43* and *C. elegans plr-1* genes showing the zinc finger domain. (**B**) Representative tracks of wild-type and *plr-1*(*tm2957*) locomotion. (**C**) Confocal images and schematic of GABAergic neurons and D-type motor neurons. (**D**) Quantification of GABAergic axon numbers in wild type and *plr-1*(*tm2957*). (**E**) Comparison of movement speed (µm/s) between wild type and *plr-1*(*tm2957*). Average axon number was quantified using 40 worms per genotype (wild type and *plr-1* mutant). Locomotion speed measurements were conducted using 45 worms per genotype.

We next monitored the mobility of wild type and *plr-1*(*tm2957*) mutants, which revealed that *plr-1*(*tm2957*) mutants display locomotion defects and might indicate neuronal defects. Similarly, the speed of *plr-1*(*tm2957*) was slower than those of the wild type. Statistical analysis showed a *p*-value = 0.000162 for this speed difference. We next examined the axonal morphology of GABAergic neurons in *plr-1*(*tm2957*) mutants. *C. elegans* had 26 GABAergic neurons. We first generated *unc-25p::GFP* labeling GABAergic neurons in *plr-1*(*tm2957*) mutants, followed by confocal microscopy analysis. Our analysis revealed an average of 17 axonal projections in wild type as compared to approximately 13.5 in *plr-1*(*tm2957*) mutants. This reduction was highly significant with a *p*-value < 2.2 × 10^−16^ (Figure 2).

**Figure 3:**
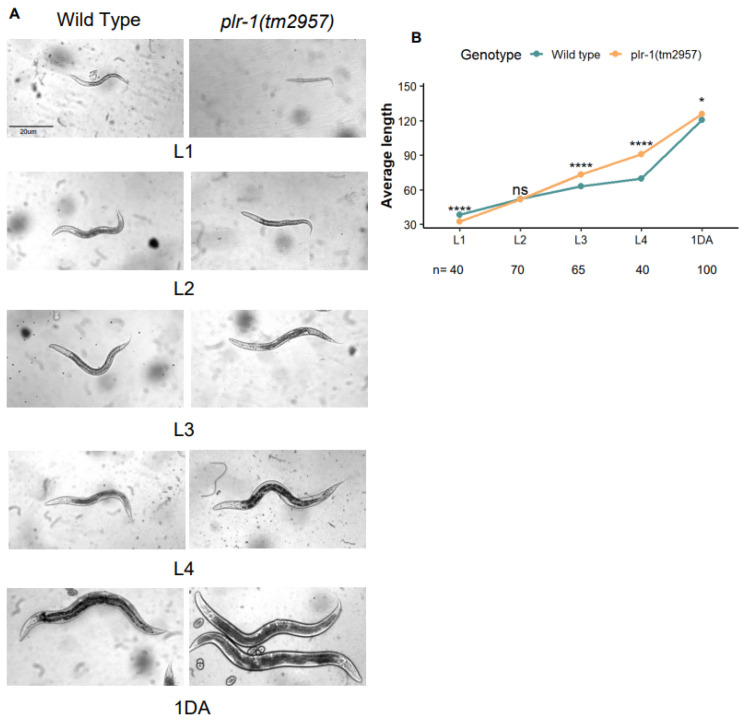
*C. elegans plr-1* mutants show differences in the developmental stages as compared to wild type. (**A**) Brightfield images of wild type and *plr-1*(*tm2957*) at L1, L2, L3, L4, and 1DA stages. (**B**) Growth curve showing average length across developmental stages. For developmental stage analysis, average body length was measured at different stages using the following sample sizes per genotype (wild type and *plr-1* mutant): L1 (n = 40), L2 (n = 70), L3 (n = 65), L4 (n = 40), and one-day-old adult (n = 100). * *p* < 0.05 and **** *p* < 0.0001.

We next examined the developmental stages of wild type and *plr-1*(*tm2957*) mutants. We found that larval 1 stage (L1) mutants were significantly shorter than the wild type. In L3 and L4 stages, the *plr-1*(*tm2957*) mutants were longer than the wild type. On day 1 (1DA), there was a mild developmental difference between wild type and *plr-1*(*tm2957*) mutants, with longer *plr-1*(*tm2957*) mutants (Figure 3).

## 3. Discussion

The present study preliminarily investigated the effect of *plr-1* mutation in the *C. elegans* model, attempting to mimic a human phenotype with neurodevelopmental abnormalities. Accordingly, *plr-1* mutation resulted in developmental delays and locomotion, as well as axonal defects in the *C. elegans* model compared to wild-type ones. Collectively, the study presents evidence suggesting that RNF43 protein may play an important role in neuronal development.

In the patient whose molecular diagnosis emphasized a biallelic NM_001305545:exon8:c.C1906T:p.Q636X variant in the *RNF43* gene, the clinical indications were developmental delay, neonatal hypotonia, frequent seizures, progressive microcephaly, and bilateral optic atrophy. This variant was listed as of unknown significance in two submissions (accession: SCV005937889.1 and SCV002268209.5) in the ClinVar database (variant accession: VCV001488568.7) [13]. In one of the submissions, the variant was speculated to cause loss of protein function putatively due to premature protein truncation or nonsense-mediated mRNA decay (NMD). Nevertheless, in the second submission in the database, NMD was not proposed since the variant eliminates the last 21 amino acids of the RNF43 protein. Collectively, both explanations point out that the consequences of this variant on the protein are not known. The variant is localized at the last exon of the *RNF43* gene, and it does not result in the elimination of the transmembrane and RING domains in the coded protein. Importantly, for patients with similar phenotypes, gain-of-function variants in another RNF gene, *RNF13*, were reported to be the underlying reason instead of loss-of-function variants [14,15]. Still, to test the results of the protein loss, which was previously shown for other genes even though the variants were localized in the last exon of the gene [16], we modeled the loss of protein function in *C. elegans*.

As a regulator of Wnt signaling by promoting ubiquitination and internalization of Frizzled receptors, RNF43 is a transmembrane E3 ubiquitin ligase [1,2]. The Wnt signaling pathway plays a critical role in neural development, regulating neuronal differentiation, axon guidance, and synaptic formation [9,17]. Dysregulation of Wnt signaling has been associated with multiple neurodevelopmental disorders including microcephaly and epilepsy [10], although RNF43 protein was not directly associated with neurodevelopmental stages. Moreover, the present study did not further evaluate the involvement of Wnt pathway molecularly, which limits the direct evidence of affected Wnt pathway as a result of mutation in the *RNF43* gene.

Our functional analyses using *C. elegans* demonstrated that mutation of the human *RNF43* ortholog *plr-1* leads to locomotion defects and reduced GABAergic axon projections. These findings are consistent with previous studies demonstrating that Wnt signaling regulates axon guidance and neuronal polarity in *C. elegans* [11,12]. Importantly, in a similar study in which mutations of *plr-1* were evaluated in terms of neuronal development in *C. elegans*, axonal defects in neurons have been reported, although these studies did not report any findings related to GABAergic axon projections [18].

In the present study, axon measurements were performed at the L4 stage, representing a late larval developmental stage rather than adulthood. Therefore, the observed defects are unlikely to result from age-dependent neuronal maintenance. Moreover, analysis across multiple stages would further strengthen the conclusion. However, the presence of the phenotype prior to adulthood supports a developmental contribution. Consistently, body length measurements across developmental stages revealed early and persistent differences between wild-type and mutant animals. Although the magnitude of change at early stages was modest, the differences were consistent and statistically significant, and even subtle alterations can be biologically meaningful in *C. elegans*.

Although the nervous system of *C. elegans* is considerably simpler than that of vertebrates, many molecular pathways regulating neuronal development are highly conserved across species. Therefore, the conserved neuronal phenotypes observed in *plr-1* mutants support the biological plausibility that RNF43 disruption may contribute to neuronal developmental abnormalities in humans.

Taken together, our findings expand the phenotypic spectrum of RNF43 and suggest that RNF43 may represent a previously unrecognized neurodevelopmental gene whose dosage imbalance affects both tumor predisposition and neuronal development.

## 4. Materials and Methods

### 4.1. RNF43 Gene Mutation

This mutation had been detected in three male children of a consanguineous couple referred to the Department of Medical Genetics. The common findings detected in the siblings were developmental delay, neonatal hypotonia, frequent seizures, progressive microcephaly, and bilateral optic atrophy. The genetic analysis revealed the *RNF43* gene mutation (NM_001305545:exon8:c.C1906T:p.Q636X).

### 4.2. Strain Maintenance

*C. elegans* strains were maintained and cultured under standard laboratory conditions at 20 °C on nematode growth medium (NGM) agar plates seeded with *Escherichia coli* OP50 as a food source, as previously described [19]. The Bristol N2 strain was used as the wild-type control. The mutant strain *plr-1*(*tm2957*) was obtained from Shigen Mitani through the National BioResource Project (NBRP). Depending on the experimental design, animals were analyzed either at the young-adult or adult stage. All animals were age-matched for the respective assays. Specifically, axon number quantifications were performed using L4 stage worms, whereas locomotion speed measurements were conducted using one-day-old adult worms.

### 4.3. Behavioral Assays

All behavioral experiments were performed on NGM plates in the absence of food using young adult hermaphrodites. To assess locomotion behavior, worms were placed in the center of an NGM plate. Following transfer, worms were allowed to acclimate for 30 s, after which their movement was recorded for 1 min.

Locomotion tracking and quantitative analyses were performed using ImageJ v1.54s with the WormTrack plugin, an automated worm tracking system used to quantify movement parameters. Each experiment was performed with at least three biological replicates.

### 4.4. Confocal Microscopy

To visualize GABAergic neurons, the transgenic strain juIs76 II [unc-25p::GFP + lin-15(+)] was used. This transgene was crossed into the *plr-1*(*tm2957*) mutant background to generate the corresponding mutant strain. Axonal morphology and axon numbers were analyzed using confocal microscopy.

For imaging, worms were mounted on 2% agarose pads containing 50 mM levamisole for immobilization. Briefly, a drop of molten agarose was placed on a glass slide and flattened with a second slide to generate an agarose pad. After solidification, 3 µL of levamisole was added and 10–15 worms were transferred onto the pad and then covered with a coverslip. Fluorescence imaging was performed using a Zeiss Airyscan 2 LSM900 confocal microscope (Carl Zeiss Microscopy GmbH, Jena, Germany) equipped with ZEN 3 Blue edition software. Worms were initially located using a 5× objective, and high-resolution images were acquired using a 20× objective with immersion oil. Z-stack images were collected and processed using ImageJ/Fiji software v1.54s for analysis. Wild-type and mutant animals were compared to quantify differences in axon number and neuronal morphology.

### 4.5. Stages and Measurements

For reliable and comparable developmental analyses of *C. elegans*, worms at the same developmental stages were used in all experiments. To obtain synchronized populations, adult hermaphrodites from wild-type (N2) and *plr-1*(*tm2957*) mutant strains were transferred to fresh NGM plates and allowed to lay eggs for 1–3 h. After laying eggs, adult worms were removed from the plates. Eggs were incubated at 20 °C, and developmental stages were monitored according to the standard developmental timing described in WormAtlas. Under these conditions, larvae reached the L1 stage approximately 12 h after hatching, the L2 stage after an additional 12 h, the L3 stage after approximately 8 h, and the L4 stage after another 8 h, and developed into 1-day-old adults after approximately 18 h. Synchronized worms were analyzed and measured at the L1, L2, L3, L4, and 1-day adult stages to determine their average body lengths. Body length measurements were performed using a compound microscope equipped with the Andor microscopy application, and images were processed and quantified using ImageJ/Fiji software.

### 4.6. Statistical Analysis

All quantitative data were analyzed using R software. Data distribution was assessed for normality before statistical testing. For normally distributed data, comparisons between two groups were performed using Student’s *t*-test, while comparisons among multiple groups were performed using one-way analysis of variance (ANOVA) followed by Tukey’s HSD post hoc test. For data that did not follow a normal distribution, the Kruskal–Wallis test followed by Dunn’s post hoc test were used. Statistical significance was calculated for measurements including axon number, locomotion speed, and developmental body length. Significance levels are indicated in the figures as asterisks. A *p*-value of < 0.05 was considered statistically significant.

### 4.7. Ethical Statement

Written informed consent was obtained from all participants prior to enrollment in the study, in accordance with the principles of the Declaration of Helsinki. According to current ethical guidelines, studies involving *C. elegans* do not require approval from an institutional ethics committee, as this organism is an invertebrate and is not subject to regulations governing the use of vertebrate animals in research.

## 5. Conclusions

Regarding the cellular and phenotypic results of a suspicious variant detected in patients, *C. elegans* could be a useful organism to follow the influences of the variant. In the present study, we focused on a homozygous variant in RNF43 gene detected in a patient with neurodevelopmental anomalies. To model the variant causing loss-of-protein function by early truncation, we investigated the developmental and neurodevelopmental features in *C. elegans* with *plr-1(tm2957*) mutation. Accordingly, the worms displayed both developmental and neurodevelopmental defects. However, variant-specific evaluations and models may alter the findings of the present study, which requires further molecular studies.

## Data Availability

The data presented in this study are available on request from the corresponding author since the data are not publicly available due to privacy or ethical restrictions.

## Abbreviations

The following abbreviations are used in this manuscript:

RNF43	Ring finger protein 43
GABA	Gamma aminobutyric acid
PLR-1	Polarity defective 1
